# Rapid and Accurate *Campylobacter jejuni* Detection With CRISPR-Cas12b Based on Newly Identified *Campylobacter jejuni*-Specific and -Conserved Genomic Signatures

**DOI:** 10.3389/fmicb.2021.649010

**Published:** 2021-04-27

**Authors:** Yu Huang, Dan Gu, Han Xue, Jinyan Yu, Yuanyue Tang, Jinlin Huang, Yunzeng Zhang, Xinan Jiao

**Affiliations:** ^1^Jiangsu Co-innovation Center for Prevention and Control of Important Animal Infectious Diseases and Zoonoses, Yangzhou University, Yangzhou, China; ^2^Jiangsu Key Laboratory of Zoonosis, Yangzhou University, Yangzhou, China; ^3^Joint International Research Laboratory of Agriculture and Agri-product Safety of the Ministry of Education, Yangzhou University, Yangzhou, China; ^4^Key Laboratory of Prevention and Control of Biological Hazard Factors (Animal Origin) for Agrifood Safety and Quality, Ministry of Agriculture of China, Yangzhou University, Yangzhou, China

**Keywords:** *Campylobacter jejuni*, CRISPR-Cas12b, protospacer discovery, comparative genomics, visualized detection

## Abstract

*Campylobacter jejuni* is among the most prevalent foodborne zoonotic pathogens leading to diarrheal diseases. In this study, we developed a CRISPR-Cas12b-based system to rapidly and accurately detect *C. jejuni* contamination. Identification of *C. jejuni*-specific and -conserved genomic signatures is a fundamental step in development of the detection system. By comparing *C. jejuni* genome sequences with those of the closely related *Campylobacter coli*, followed by comprehensive online BLAST searches, a 20-bp *C. jejuni*-conserved (identical in 1024 out of 1037 analyzed *C. jejuni* genome sequences) and -specific (no identical sequence detected in non-*C. jejuni* strains) sequence was identified and the system was then assembled. In further experiments, strong green fluorescence was observed only when *C. jejuni* DNA was present in the system, highlighting the specificity of this system. The assay, with a sample-to-answer time of ∼40 min, positively detected chicken samples that were contaminated with a dose of approximately 10 CFU *C. jejuni* per gram of chicken, which was >10 times more sensitive than the traditional *Campylobacter* isolation method, suggesting that this method shows promise for onsite *C. jejuni* detection. This study provides an example of bioinformatics-guided CRISPR-Cas12b-based detection system development for rapid and accurate onsite pathogen detection.

## INTRODUCTION

The foodborne pathogen *Campylobacter jejuni* has been recognized as the leading cause of bacterial diarrheal disease in many areas across the world (Burnham and Hendrixson, 2018), and can also cause bacteremia, sepsis, and Guillain–Barré syndrome among other diseases (Kaakoush et al., 2015). Consumption of contaminated poultry products, such as chicken, is the main cause of *C. jejuni* infection (Allos, 2001). Therefore, detection of *C. jejuni* contamination in poultry products is critical for food safety and public health. The conventional bacterial culture-based methods can confirm the pretense of *C. jejuni* contamination based on the isolated strains and are thus recognized as the “gold standard” for diagnosis of *C. jejuni* contamination (de Boer et al., 2015). However, the bacterial isolation process is time-consuming (at least 2 days are needed), and thus is unsuitable for on-site rapid pathogen detection, and is also labor-intensive because it is difficult to distinguish *C. jejuni* contaminated and uncontaminated samples in advance. For instance, data collected from more than 20 provinces in China spanning several years revealed that the detection rate of *C. jejuni* in raw chicken was 0.29–2.28% (Wang et al., 2013). Hence, it is important to develop cultivation-independent detection methods to identify *C. jejuni* contaminated samples rapidly, which will also be meaningful for the pathogen isolation process. Several types of cultivation-independent *C. jejuni* detection methods, such as antibody-based (Masdor et al., 2016; He et al., 2018; Wang et al., 2018), PCR-based (de Boer et al., 2015; Sabike et al., 2016; Liang et al., 2018; Geng et al., 2019), probe hybridization-based (Donatin et al., 2013), as well as other methods (Velusamy et al., 2010), have been developed in recent years. However, the majority of these procedures are impracticable for on-site real-time detection of *C. jejuni* because specialized and expensive equipment, such as PCR thermal cyclers, and specialized skills are required to perform the detection procedures.

Recently, the RNA-guided clustered regularly interspaced short palindromic repeats–CRISPR-associated (CRISPR-Cas) method has shown considerable promise for rapid nucleic acid detection owing to its high reliability and specificity (Li et al., 2019b). Several CRISPR-Cas-based detection systems, such as SHERLOCK (Gootenberg et al., 2017), DETECTR (Chen et al., 2018), HOLMES (Li et al., 2018), and CDetection (Teng et al., 2019), have been developed, with several types of Cas nucleases, such as Cas12a, Cas12b, and Cas13a, employed in these systems. Cas12b (C2c1) is a dual-RNA-guided DNA endonuclease that shows collateral cleavage activity after it is activated by the single-molecule guide RNA (sgRNA)–target complex. In this process, Cas12b recognizes the complex at single nucleotide-level discrimination, and then its surrounding non-target single-stranded DNAs (ssDNAs) are cleaved (Liu et al., 2017; Li et al., 2019a). Based on this property, CRISPR-Cas12b-based nucleic acid detection systems, such as HOLMES v2 and CDetection (Li et al., 2019a; Teng et al., 2019), were developed, which have shown strong practical potential for rapid, sensitive, and specific pathogen detection (Wang et al., 2020).

With the rapidly increasing number of bacterial genome sequences accessible publicly, identification of specific and conserved genomic signatures for a given bacterial population by means of comparative genomic analyses is feasible. In this study, we identified *C. jejuni*-specific and -conserved genomic signatures, and developed a CRISPR-Cas12b-based *C. jejuni* detection system in which the identified signatures were used to design the sgRNA. The developed CRISPR-Cas12b-based *C. jejuni* detection system showed high sensitivity and specificity for *C. jejuni* detection based on extracted DNA samples, and artificially contaminated chicken samples as well as retailed chicken and the associated environmental samples collected from a retail market. The procedure accepts crude DNA prepared by boiling, no bacterial isolation or DNA purification is needed, and the sample-to-answer time is ∼40 min; thus, it is a promising approach for onsite *C. jejuni* detection.

## Materials and Methods

### Strains and Reagents

*Alicyclobacillus acidoterrestris* was purchased from the Agricultural Culture Collection of China and was used for Cas12b (AacCas12b) gene amplification. The strains *C. jejuni* NCTC11168, NCTC81-176 and YZ1, *Campylobacter coli* YZ2, *Escherichia coli* ATCC 25922, *Salmonella* Enteritidis C50041, *S.* Typhimurium SL1344, *S.* Derby YZ14, *S.* Dublin CMCC 50042, *Vibrio parahaemolyticus* RIMD2210633, *Staphylococcus aureus* ATCC27217, and *Listeria monocytogenes* EGD-e were used for CRISPR-Cas12b-based *C. jejuni* detection system evaluation. Strains with the YZ prefix were isolated by our laboratory.

DNA oligonucleotides and ssDNA-FQ fluorescent probes were synthesized by GenScript Biotechnology (Nanjing, China).

### AacCas12b Protein Purification

The full-length sequence of the Cas12b-encoding gene was amplified, cloned into the pET-28a vector, and transformed into *E. coli* strain BL21 (DE3) as described previously (Li et al., 2019a). Expression of the Cas12b protein was induced by addition of 0.5 mM isopropyl-1-thio-β-D-galactopyranoside and incubation for 18–24 h at 15°C. Cell pellets were resuspended in lysis buffer containing 50 mM Tris–HCI (pH 7.6), 150 mM NaCl, 20 mM imidazole, and 1/500 phenylmethylsulfonyl (v/v), then lysed with a 30-W ultrasonic cell disruptor for 10 min (sonicating for 3 s per cycle with a time interval of 5 s), and centrifuged at 11,000 × *g* for 15 min. The supernatant was filtered through a 0.22-μm filter and purified using the His Bind Purification Kit (Novagen). The eluted protein was concentrated using Millipore concentrators and validated using SDS-PAGE and Western blotting. The concentration of the obtained protein was quantified using the BCA Protein Assay Kit (Beyotime).

### *C. jejuni*-Specific Genomic Signature Identification and sgRNA Preparation

All *C. jejuni* and *C. coli* genome sequences accessible in the NCBI refseq database (as of November 21, 2018) were downloaded. The representative and reliable *C. jejuni* and *C. coli* genome sequences were retained if the genome conformed to the following two criteria: (i) the genome-wise average nucleotide identity (ANI) value between the downloaded genome and the *C. jejuni*-type strain NCTC11351 (refseq ID GCF_001457695.1) or *C. coli*-type strain NCTC 11366 (refseq ID GCF_900446355), calculated using FastANI (Jain et al., 2018), was higher than the species demarcation threshold of 95% (Richter and Rossello-Mora, 2009); and (ii) the genome harbored *mapA* or *ceuE* genes and the corresponding genes could be *in silico* amplified (Stucki et al., 1995; Gonzalez et al., 1997; Wieczorek and Osek, 2005) using the Seqkit amplicon algorithm (Shen et al., 2016). Sequence types of the genomes were determined using MLST software (ver. 2.19.0) (Seemann, unpublished) based on the PubMLST database (Jolley and Maiden, 2010). The representative strains from each ST were selected and applied to Neptune software (parameter –size 30) (Marinier et al., 2017) for *C. jejuni*-specific sequence identification. Briefly, the genome sequences of those selected *C. jejuni* and *C. coli* strains were splitted to shorter sequence signatures and the signatures were compared between each other, and the sequence signatures that were sufficiently common to the *C. jejuni* group and sufficiently absent to the *C. coli* group with length longer than 30 bp were extracted for further analysis (Marinier et al., 2017). The identified *C. jejuni*-specific sequence signatures were blasted against the 1037 *C. jejuni* genomes using local BLASTn and against the non-*C. jejuni* genome sequences using the NCBI online BLAST tool to check the specificity of the signature sequences.

A representative *C. jejuni*-specific signature sequence M1 was selected, and its corresponding protospacer target T1 was designed and synthesized accordingly. T1 was cloned into the pUC18 vector and then amplified using the T7-sgRNA-F and Target-T1-R primers. The quantified purified PCR product was used as the template for sgRNA biosynthesis using the T7 High Yield Transcription Kit (Thermo Fisher) by incubation at 37°C for 12 h. DNase I was added to the reaction solution and incubated at 37°C for 30 min to digest the remaining DNA fragments. The transcribed sgRNA G1 was purified using the RNA Clean & Concentrator TM-5 Kit (Zymo Research) and stored at −80°C.

### Target Cleavage Activity Determination of the Cas12b Protein and Detection System Assembly

To verify the *trans*- and *cis*-cleavage activity of the purified Cas12b protein (Li et al., 2019a), a genomic region containing T1 of *C. jejuni* NCTC81-176 was amplified using the primer set Kong-F1 and Kong-R1 (Table 1). The size of the PCR product was ∼1000 bp and T1 was located at position ∼400 bp in the product. PCR assays were performed using a T100 Thermal Cycler (Eppendorf) with an initial denaturation step at 98°C for 3 min, followed by 30 cycles at 98°C for 30 s, 55°C for 30 s, and 72°C for 1 min, and terminated with a final extension at 72°C for 10 min. The PCR product was mixed with the Cas12b protein in the reaction buffer, incubated at 48°C for 30 min, and then 3 μl of 6 × Cas-STOP Loading Buffer was added and incubated at 65°C for an additional 5 min before separation by gel electrophoresis.

**TABLE 1:** Oligonucleotides used in this study.

**Oligo names**	**Sequences (5′-3′)**
M1	GTTTGCCTCAGCAATAACTTCTTGACGTCTTGCTCTAGCGG TTTGT
Target T1	TTGACGTCTTGCTCTAGCGG
T7-sgRNA-F	GAAATTAATACGACTCACTATAGGG
Target-T1-R	CCGCTAGAGCAAGACGTCAAGTGCCACTTCTCAGATTTGA GAAG
sgRNA G1	GAAAUUAAUACGACUCACUAUAGGGGUCUAGAGGACAGAA UUUUUCAACGGGUGUGCCAAUGGCCACUUUCCAGGUGGC AAAGCCCGUUGAGCUUCUCAAAUCUGAGAAGUGGCACUU GACGUCUUGCUCUAGCGG
Kong-F1	GCGTGTGAGAAGTTCGCTTG
Kong-R1	TCTTGGGGCCTTTAATCGCT
Kong-F2	CACCTGCTCCACTTTGAGATG
Kong-R2	TCTTGGGGCCTTTAATCGCT

*The underlined sequences are the newly identified *C. jejuni*-conserved and -specific protospacer T1.*

The CRISPR-Cas12b-based *C. jejuni* detection system predominantly contained the Cas12b protein, sgRNA G1, sample DNA, ssDNA-FQ fluorescent probe 5′-^6^FAM-N12-3′-BHQ1, recombinant RNase inhibitor, buffer, and RNase-free water. When the sample DNA matched the target T1, the presence of strong green fluorescence signal was assessed by the naked eye under 485-nm light.

### Specificity and Sensitivity Evaluation of the CRISPR-Cas12b-Based *C. jejuni* Detection System

To evaluate the specificity of the CRISPR-Cas12b system for *C. jejuni* detection, the DNA samples of *C. jejuni* and its close relative *C. coli*, as well as strains of *Salmonella*, *V. parahaemolyticus*, *E. coli*, and the other aforementioned foodborne pathogenic bacteria were employed as targets. The DNA samples were added to the detection system individually or mixed, and the presence of green fluorescence signal for each reaction system was assessed under 485-nm light by the naked eye.

To evaluate the sensitivity of the CRISPR-Cas12b-based *C. jejuni* detection system, a 1559-bp genomic region containing the protospacer target T1 was amplified using the primer set Kong-F2 and Kong-R2 (Table 1). PCR assays were performed using a T100 Thermal Cycler (Eppendorf) with an initial denaturation step at 98°C for 3 min followed by 30 cycles at 98°C for 30 s, 55°C for 30 s, and 72°C for 90 s, and terminated with a final extension at 72°C for 10 min. The initial concentration of the purified PCR product was 111 ng/μl, as measured using a NanoDrop spectrophotometer, and the corresponding copy number was calculated (Zhang et al., 2020). The PCR product was diluted in 1:10 serial dilutions, and 3 μl was added to the detection system as the DNA template. The presence of green fluorescence for each dilution was assessed under 485-nm light by the naked eye and the fluorescence intensity was determined using ImageJ software (Jensen, 2013). *C. jejuni* genomic DNA was extracted using the TIANamp Bacteria DNA Kit (Tiangen Biotech). The initial concentration of the genomic DNA was 204 ng/μl. The genomic DNA was diluted, added to the detection system, and the presence and intensity of the fluorescence was determined as described above.

### Evaluation of the CRISPR-Cas12b-Based *C. jejuni* Detection System Based on the Chicken Samples With Spiked-in *C. jejuni* Contamination

Frozen chicken was purchased from a local supermarket in Yangzhou and chopped to approximately 25-g pieces. The chopped chicken samples were placed in a biological safety hood under ultraviolet light for 30 min, and then washed with ddH_2_O twice to eliminate the potential effects of chicken-carried *C. jejuni* on the detection process. The cleaned chicken samples were air-dried and then immersed in tubes filled with a graded series of *C. jejuni* concentrations, and incubated for 1 h under ambient temperature (42 ± 1°C). The initial suspension of *C. jejuni* was OD_600_ = 1 and was diluted in 1:10 serial dilutions. The chicken samples were removed from the bacterial suspension, dried using sterilized filter paper, and then added to sample collection bags that were filled with 225 ml of PBS. The samples were manually kneaded for 1 min. Bacterial isolation was performed as described for *C. jejuni* GB 4789.9e2014 for the microbiological investigation of food hygiene (National Food Safety Standards of China). One milliliter of the solution was placed in a 1.5-ml centrifuge tube and centrifuged at 8000 × *g* for 5 min. The pelleted bacterial cells were washed once with PBS buffer and resuspended with 100 μl of RNase-free water. Finally, the suspensions were boiled at 100°C for 5 min and centrifuged at 8000 × *g* for 2 min. The resultant supernatant was used as input template DNA for the detection system.

### Evaluation of the CRISPR-Cas12b-Based *C. jejuni* Detection System Based on the Chicken and Environmental Samples Collected From a Retail Market

To evaluate the practical efficiency of our newly developed CRISPR-Cas12b-based *C. jejuni* detection system, environmental and chicken samples were collected from a retail market in Yangzhou, Jiangsu, China in December 2020. In this market, live chickens that were fed by rural farmers were slaughtered and sold on demand. Wiping samples were collected from environmental samples including knife, floor, stool, and cages, as well as chicken samples including the whole chicken surface of the dehaired carcasses, and half exterior surface and half interior surface of the eviscerated carcasses by phosphate buffer (PBS, pH 7.2) immersed with sterilized cotton balls as described by Tang et al. (2020). A total of 118 samples, including 63 environmental samples and 55 chicken wiped samples, were collected. Each sample was 1:10 diluted using PBS buffer, and then 1 ml of the diluted solution was placed in a 1.5-ml centrifuge tube and centrifuged at 8000 × *g* for 5 min. The pelleted bacterial cells were washed once with PBS buffer and resuspended with 100 μl of RNase-free water. Finally, the suspensions were boiled at 100°C for 5 min and centrifuged at 8000 × *g* for 2 min. The resultant supernatant was used as input template DNA for the detection system. Bacterial isolation was also performed as described above.

## Results and Discussion

### Identification of *C. jejuni*-Specific and -Conserved Genomic Signatures Through Comparative Genomic Analysis

Given the extremely harmful impact of *C. jejuni* on human health, rapid identification of *C. jejuni* contamination in chicken as well as other products is vital to reduce the risk of contaminated products reaching consumers and of food poisoning caused by *C. jejuni*. Recently, several CRISPR-Cas-based methods have shown considerable promise for rapid detection of pathogens such as SARS-CoV-2 (Ding et al., 2020; Wang et al., 2020). In the present study, we sought to develop a CRISPR-Cas12b-based detection system to identify *C. jejuni* contamination rapidly and reliably. Identification of *C. jejuni*-specific and -conserved genomic signatures, which can be used as the protospacer, is a fundamental step in the process of developing a CRISPR-Cas12b-based *C. jejuni* detection system. All publicly available genome sequences annotated as *C. jejuni* in the National Center for Biotechnology Information (NCBI) refseq database were downloaded (as of November 21, 2018). Overly fragmented genomes with scaffolds >100 were discarded and 1041 *C. jejuni* genome were retained for further analysis. However, we established that the taxonomic annotations were incorrectly assigned for certain genomes; for instance, GCF_002177345 was annotated as *C. jejuni*, but the *C. jejuni* lineage-specific *mapA* gene (Stucki et al., 1995) was not identified in the GCF_002177345 genome as revealed by the BLASTn algorithm, and the genome-wide ANI value between this genome and the *C. jejuni*-type strain NCTC11351 was only 77%, which was significantly lower than the species demarcation threshold of ∼95% (Richter and Rossello-Mora, 2009). Additional 16S rDNA-based analysis demonstrated that this strain showed much higher sequence identity with *Campylobacter lari* subsp. *concheus* strain 2897R (identity = 99.73%) than with *C. jejuni* NCTC11351 (identity = 98.96%) based on the NCBI 16S_ribosomal_RNA database. Collectively, these findings were indicative of incorrect taxonomic annotation of GCF_002177345. To obtain *C. jejuni*-specific and -conserved signatures with confidence, we first discarded genome sequences that showed ANI values < 95% with the *C. jejuni*-type strain NCTC11351 and/or did not harbor the *mapA* gene. In this manner, four genome sequences were discarded accordingly.

The primer sequences that are used for amplification of pathogen-specific marker genes can be ideal candidates for protospacer identification. The *mapA* gene is known to be a *C. jejuni*-specific marker gene and no degenerate residue was present in the primer sequences (Stucki et al., 1995), and thus, we evaluated whether the *mapA* primer set can be used for protospacer discovery. *In silico* PCR analysis was then performed based on the *mapA*-specific primer set using the Seqkit amplicon algorithm (Wieczorek and Osek, 2005; Shen et al., 2016). The results demonstrated that 889 of the 1037 genomes harbored exactly the same sequences as the *mapA* gene primer set (Wieczorek and Osek, 2005). However, two kinds of single nucleotide mismatch in the *mapA*-F primer were detected for 148 genomes (accounting for 14.3% of the 1037 genomes), and these genomes were affiliated with 31 ST types (Supplementary Table 1). These mismatches did not affect the PCR reaction and the *mapA* product was successfully obtained as confirmed by PCR experiments using the strain *C. jejuni* NCTC11168 as template (data not shown). Based on the criteria for CRISPR-Cas12b protospacer discovery, only an 18-bp sequence (TATTTTTGAGTGCTTGTG) from the *mapA*-F primer was identified as a candidate protospacer, while no potential protospacer was identified from the *mapA*-R primer. The sgRNAs were synthesized based on the 18-bp sequence as well as its two mutated forms (TATTTTTGAG**C**GCTTGTG and TATTTTTGAGTGCTTG**C**G, SNPs labeled in bold). Our results demonstrated that only very weak fluorescence signals that could not be differentiated with the background noise (*p* > 0.05) were observed when the three kinds of sgRNAs were added in the CRISPR-Cas12b detection system (Supplementary Figure 1). This was consistent with a previous report that 20 bp is the ideal length for the protospacer used in the CRISPR-Cas12b detection system (Yang et al., 2016). These results suggested that the *mapA* primer sequences were not suitable for protospacer discovery.

**FIGURE 1:**
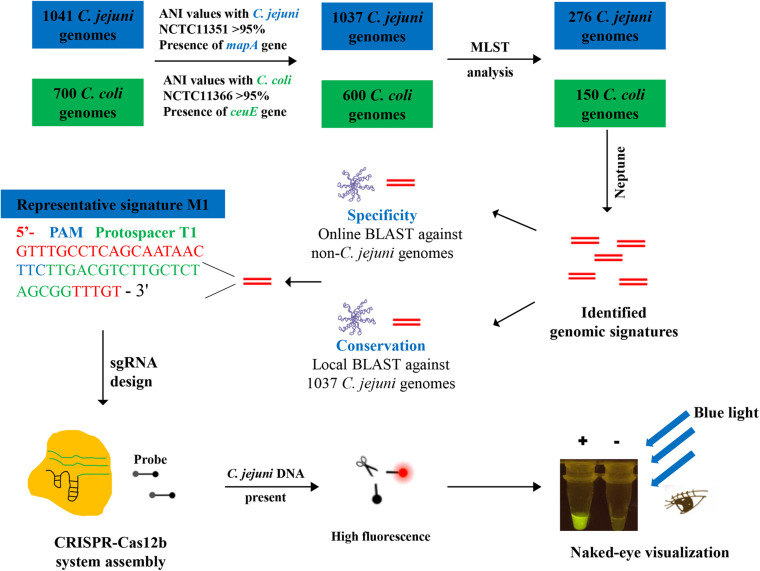
Schematic of the CRISPR-Cas12b-based *C. jejuni* detection method based on newly identified *C. jejuni*-specific and -conserved genomic signatures.

We then sought to identify *C. jejuni*-specific and -conserved genomic signatures by comparing the *C. jejuni* genomes with genomic sequences for the closely related *C. coli*. Following the same aforementioned procedures, 600 high-quality *C. coli* genome sequences were extracted from the NCBI refseq database (Supplementary Table 2). Given the computational capacity of the Neptune software (i.e., the maximum genome number accepted for each group is 400) (Marinier et al., 2017), the sequence type (ST) schemes of the remaining genomes were determined using MLST software (ver. 2.19.0) (Seemann, unpublished), and representative genomes were randomly selected from each ST type. As a result, 276 *C. jejuni* and 150 *C. coli* representative genomes were selected for signature discovery (Supplementary Tables 1,2). Fifteen signatures with a score >0.95, which was calculated as the proportion of *C. jejuni* genomes harboring a given signature (exact match) minus the proportion of *C. coli* genomes with the identical signature identified, were selected for protospacer discovery. The selected signatures should contain the Cas12b protospacer adjacent motif (PAM) sequence TTN (Jain et al., 2019) and the PAM sequence should be located in an appropriate position. Finally, a representative signature designated M1 of length 46 bp, which was located within the flagellar biosynthesis-associated *flhA* gene, was identified in 99.31% (276 out of 278) of the *C. jejuni* genomes (exact sequence match) but was not identified in a *C. coli* genome (10 mismatches compared with the sequence from *C. coli* type strain NCTC11366), and thus was used for protospacer discovery (Table 1). Online BLAST searches for M1 and its corresponding protospacer target T1 further demonstrated the specificity of M1 and T1 because no hit resulted when M1 and T1 were used as queries and the *C. jejuni*-excluded NCBI nt database was used as the subject database. The conservation of T1 in the 1037 *C. jejuni* genomes was also determined using a local BLASTn search, which demonstrated that 1024 out of the 1037 genomes harbored the T1 sequence (exact match) (Supplementary Table 1). The workflow for *C. jejuni*-specific and conserved genomic signature discovery and the CRISPR-Cas12b-based *C. jejuni* detection system assembly is shown in Figure 1.

### Assembly, Specificity, and Sensitivity Assessment of the CRISPR-Cas12b-Based *C. jejuni* Detection System

**FIGURE 2:**
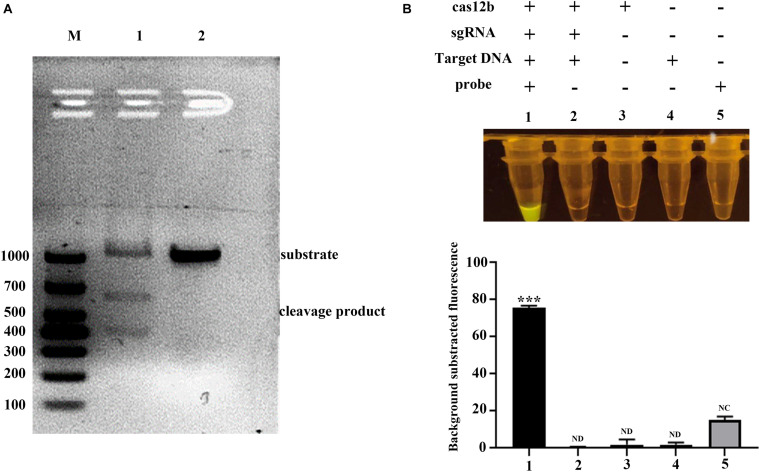
Feasibility assessment of the CRISPR-Cas12b-based *C. jejuni* detection system. **(A)** The *cis*-cleavage activity of Cas12b. Lane M, DL1000 Marker. Lane 1: cleavage by Cas12b of the PCR product. In this system, the PCR product (substrate of Cas12b), sgRNA, Cas12b, and other reagents were added. Lane 2: negative control. Cas12b was not added in the system. **(B)** The *trans*-cleavage activity of Cas12b. Upper panel, Tube 1: all required components were added; Tube 2: the 5′−^6^FAM-N12-3′-BHQ1 probe was not included; Tube 3: only Cas12b was added; Tube 4: only template DNA was added; Tube 5: only the 5′−^6^FAM-N12-3′-BHQ1 probe was added. Lower panel: fluorescence intensity corresponding to each tube in panel **(B)** upper panel. Paired two-tailed *t* test, ^∗^*p* < 0.05, ^∗∗^*p* < 0.01, ^∗∗∗^*p* < 0.001. ND, Not detected. NC, Negative control.

The CRISPR-Cas12b-based *C. jejuni* detection system was assembled, and its sensitivity and specificity were evaluated. First, we amplified a ∼1-kb sequence amplicon using the primer set Kong-F1 and Kong-R1 (Table 1) in which the protospacer target T1 was located at ∼400 bp in the PCR product. We then added the PCR product to the detection system to verify the cleavage activity of the Cas12b protein. The PCR product was cleaved into two fragments of the expected sizes (Figure 2A). When the ssDNA-FQ fluorescent probe 5′-^6^FAM-N12-3′-BHQ1 was further added, strong green fluorescence signal was observed (Figure 2B). Collectively, these results demonstrated the feasibility of the CRISPR-Cas12b-based *C. jejuni* detection system.

**FIGURE 3:**
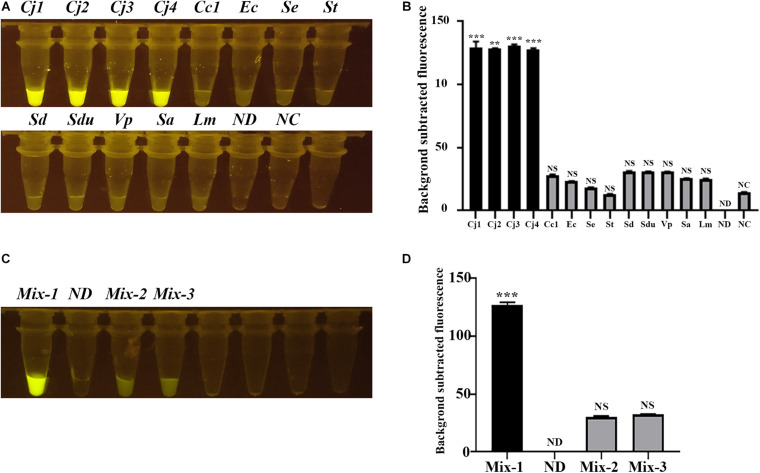
Specificity assessment of the CRISPR-Cas12b-based *C. jejuni* detection system. **(A)** Specificity analysis based on individual *C. jejuni* and non-*C. jejuni* strains. Tubes Cj1 and Cj2: *C. jejuni* NCTC 81-176; Tube Cj3: *C. jejuni* NCTC 11168; Tube Cj4: *C. jejuni* YZ1; Tube Cc1: *C. coli* YZ2; Tube Ec: *E. coli* ATCC 25922; Tube Se: *S.* Enteritidis C50041; Tube St: *S.* Typhimurium SL1344; Tube Sd: *S.* Derby YZ14; Tube Sdu: *S.* Dublin CMCC 50042; Tube Vp: *Vibrio parahaemolyticus* RIMD2210633; Tube Sa: *Staphylococcus aureus* ATCC 27217; Tube Lm: *Listeria monocytogenes* EGD-e; Tube ND: no DNA and probe were added; Tube NC: only the 5′-6FAM-N12-3′-BHQ1 probe was added. **(B)** Fluorescence intensity corresponding to each tube in panel **(A)**. **(C)** Specificity analysis based on mixed DNA samples. Tube Mix-1: mixed DNA from the three *C. jejuni* strains; Tube ND: no DNA and probe were added; Tube Mix-2: mixed DNA from all the non-*Campylobacter* strains mentioned above; Tube Mix-3: mixed DNA from all the non-*C. jejuni* strains mentioned above. **(D)** Fluorescence intensity corresponding to each tube in panel **(C)**. Paired two-tailed *t* test, ^∗^*p* < 0.05, ^∗∗^*p* < 0.01, ^∗∗∗^*p* < 0.001. NS, Not significant. ND, Not detected. NC, Negative control.

We then selected several *C. jejuni* strains and other related zoonotic bacterial pathogens, including *C. coli*, which is closely related to *C. jejuni*, and *E. coli*, *Salmonella* Enteritidis, *S.* Typhimurium, *S.* Derby, *S.* Dublin, *V. parahaemolyticus*, *S. aureus*, and *L. monocytogenes*, to evaluate the specificity of the CRISPR-Cas12b-based *C. jejuni* detection system. The tubes with *C. jejuni* DNA present showed strong green fluorescence signal, whereas no obvious green fluorescence signal was observed for other tubes containing DNA from non-*C. jejuni* strains (Figures 3A,B). When the DNA from different strains were mixed, only the tube with *C. jejuni* DNA present exhibited strong fluorescence signal (Figures 3C,D). These results clearly demonstrated that the CRISPR-Cas12b-based *C. jejuni* detection system recognized specifically *C. jejuni*-derived DNA in the DNA samples owing to the single nucleotide-level discrimination ability of CRISPR-Cas12b (Li et al., 2019a).

**FIGURE 4:**
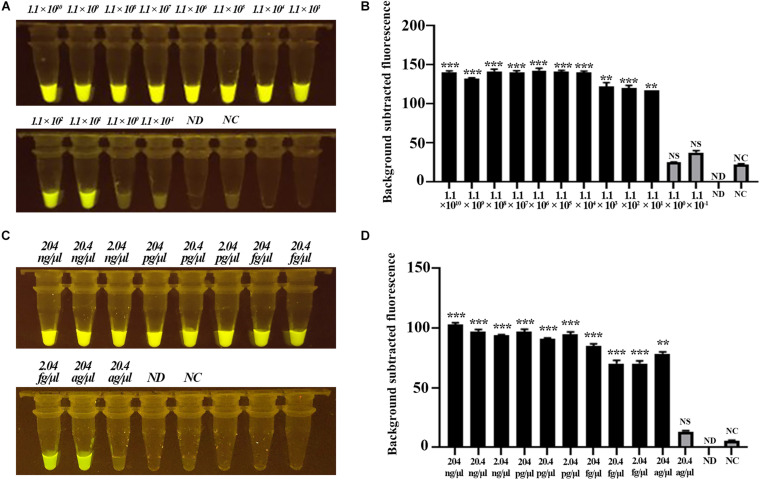
Sensitivity assessment of the CRISPR-Cas12b-based system for *C. jejuni* detection. **(A)** Sensitivity analysis based on 10-fold gradient dilution of the target T1-containing PCR product. Tubes 1–12: PCR product with concentration from 1.1 × 10^10^ copies/μl to 1.1 × 10^– 1^ copies/μl; Tube ND: no DNA and probe were added; Tube NC: only the 5′−^6^FAM-N12-3′-BHQ1 probe was added. **(B)** Fluorescence intensity corresponding to each tube in panel **(A)**. **(C)** Sensitivity analysis based on 10-fold gradient dilution of *C. jejuni* genomic DNA. Tubes 1–11: genomic DNA concentration from 204 ng/μl to 20.4 ag/μl. Tube ND: no DNA and probe were added; Tube NC: only the 5′−^6^FAM-N12-3′-BHQ1 probe was added. **(D)** Fluorescence intensity corresponding to each tube in panel **(C)**. Paired two-tailed *t* test, ^∗^*p* < 0.05, ^∗∗^*p* < 0.01, ^∗∗∗^*p* < 0.001. NS, Not significant. ND, Not detected. NC, Negative control.

Next, we evaluated the sensitivity of the CRISPR-Cas12b-based *C. jejuni* detection system based on gradient-diluted target T1-containing PCR product as well as the *C. jejuni* genomic DNA. The initial concentration of PCR product was 111 ng/μl, equivalent to 1.1 × 10^10^ copies of nucleotide sequence per microliter, and the initial concentration of *C. jejuni* DNA was 204 ng/μl. The PCR product and genomic DNA were 10-fold gradient-diluted, and 3 μl of PCR product or genomic DNA was added to the detection system. Strong green fluorescence signal was observed for samples at concentrations ranging from 1.1 × 10^10^ to 1.1 × 10^1^ copies/μl for the PCR product-based assay and 204 ng/μl to 204 ag/μl for the genomic DNA-based assay. These results indicated that the detection limit of the CRISPR-Cas12b-based *C. jejuni* detection system was <11 copies or <204 ag/μl genomic DNA per reaction (Figure 4). The sensitivity of the CRISPR-Cas12b-based detection system was comparable with that of previous loop-mediated isothermal amplificatio]n (LAMP) and real-time PCR-based *C. jejuni* detection methods (Leblanc-Maridor et al., 2011; Zang et al., 2017). However, no specialized equipment, such as a PCR thermal cycler, is required and the present assay requires a much shorter detection period.

### Feasibility Assessment of the CRISPR-Cas12b-Based *C. jejuni* Detection System for Onsite *C. jejuni* Detection

**FIGURE 5:**
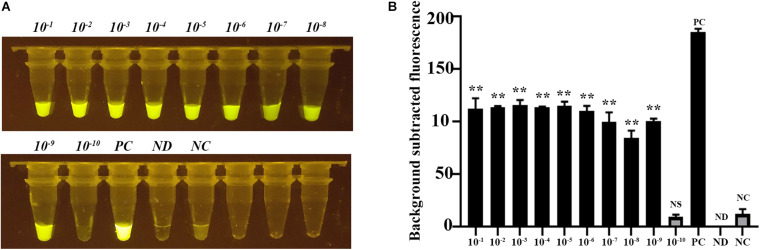
Feasibility assessment of the CRISPR-Cas12b-based *C. jejuni* detection system for onsite *C. jejuni* detection based on chicken samples contaminated with different doses of *C. jejuni*. **(A)** Chicken samples (25 g) detected with the CRISPR-Cas12b system; Tubes 1–10: crude DNA extracted from chicken samples contaminated with 10-fold gradient dilutions of *C. jejuni* NCTC81-176 with an initial concentration of OD_600_ = 1; Tube PC: positive sample; Tube ND: no DNA and probe were added; Tube NC: only the 5′−^6^FAM-N12-3′-BHQ1 probe was added. **(B)** Fluorescence intensity corresponding to each tube in panel **(A)**. Paired two-tailed *t* test, ^∗^*p* < 0.05, ^∗∗^*p* < 0.01, ^∗∗∗^*p* < 0.001. NS, Not significant. PC, Positive control. ND, Not detected. NC, Negative control.

We assessed the feasibility of the CRISPR-Cas12b-based *C. jejuni* detection system for onsite *C. jejuni* detection using chicken samples contaminated with 10-fold gradient dilutions of *C. jejuni* NCTC81-176 with an initial concentration of OD_600_ = 1. The contaminated chicken samples were washed using PBS buffer, and then DNA was extracted from the wash buffer by boiling for 5 min. The crude DNA extracted from chicken samples contaminated with 10^–1^ OD to 10^–9^ OD of *C. jejuni* showed strong green fluorescence signal (Figures 5A,B). Further *C. jejuni* isolation and identification results demonstrated that no *C. jejuni* colony was formed for the sample from the 10^–8^ OD dilution, suggesting that the CRISPR-Cas12b-based detection system was ∼10 times more sensitive than the bacterial isolation approach. Further plate-counting results revealed that the bacteria load for chicken samples contaminated with 10^–7^ OD was approximately 1000 CFU/g chicken, and thus the detection limit of the CRISPR-Cas12b-based *C. jejuni* detection system was ∼10 CFU/g chicken sample. This detection limit was significantly lower than the critical limit of *C. jejuni* contamination in chicken samples (1000 CFU/g sample) as proposed by the European Food Safety Authority [Commission Regulation (EU) 2017/1495]. Thus, the sensitivity of the CRISPR-Cas12b-based *C. jejuni* detection system is acceptable, and the application of DNA amplification methods such as recombinase polymerase amplification and LAMP (Li et al., 2019a; Williams et al., 2019) is not required to save time and reduce the cost. Of note, the CRISPR-Cas12b-based *C. jejuni* detection system is activated and strong fluorescence signal is observed when the dose of *C. jejuni* in the detected chicken sample is higher than 10 CFU/g chicken sample (Figure 5), and thus the result is qualitative but not quantitative. Further quantity-aware methods such as conventional *C. jejuni* isolation can be performed for the CRISPR-Cas12b-positive samples to determine the exact dose of *C. jejuni* in the given samples.

Finally, we applied the CRISPR-Cas12b-based *C. jejuni* detection system to detect *C. jejuni* contamination in retailed chicken samples and the associated environmental samples. In this kind of retail market, the chicken meat is frequently contaminated with low levels of *Campylobacter*, and the associated environment is also easily cross-contaminated, resulting in a high contamination rate of *Campylobacter* in the chicken and the associated environmental samples (Walker et al., 2019). Our results demonstrated that 82.2% of the collected samples (97/118) were *C. jejuni*-positive as suggested by the CRISPR-Cas12b-based *C. jejuni* detection method, while 81 of the 97 CRISPR-Cas12b-positive samples (83.5%) and none of the CRISPR-Cas12b-negative samples were identified as *C. jejuni*-positive by the conventional bacterial isolation-based method (Supplementary Figure 2). Furthermore, three of the 118 samples were identified as *C. coli*-positive by the conventional bacterial isolation-based method, with none of the three samples exhibiting *C. jejuni*-positive results by the CRISPR-Cas12b-based *C. jejuni* detection system. These results suggested that the CRISPR-Cas12b-based *C. jejuni* detection system was promising for onsite *C. jejuni* detection.

## Conclusion

In this study, we identified *C. jejuni*-specific and -conserved genomic signatures and developed a CRISPR-Cas12b-based *C. jejuni* detection system that shows high specificity and sensitivity with a relatively short detection time (∼40 min). Thus, this method is a promising approach for onsite *C. jejuni* detection. Our study provides an example of the development of a rapid and accurate CRISPR-Cas12b-based detection method in which the protospacer is discovered by comparing the genome sequence of the target pathogen with the genomes from a closely related taxon followed by comprehensive online BLAST searches.

## Data Availability Statement

The original contributions presented in the study are included in the article/Supplementary Material, further inquiries can be directed to the corresponding author/s.

## Author Contributions

YZ and XJ administered the project and reviewed and edited the draft. YZ proposed the concept. YH, DG, HX, JY, YT, and YZ designed and performed the experiments. YT and JH provided the resources. JY showed the visualization. YH, DG, and YZ wrote the original draft. All authors contributed to the article and approved the submitted version.

## Conflict of Interest

The authors declare that the research was conducted in the absence of any commercial or financial relationships that could be construed as a potential conflict of interest.

## Supplementary Material

Supplementary Figure 1 Feasibility Assessment of the candidate protospacers identified from the *mapA* primer sequences. **(A)** Fluorescence signals in the CRISPR-Cas12b-based detection system when different candidate protospacers were added in the system. Tube target T1:sgRNA based on protospacer target T1 was added; Tube map-F1: sgRNA based on the 18 bp protospacer which was conversed in 889 genomes, map-F1 (TATTTTTGAGTGCTTGTG) was added; Tube map-F2: sgRNA based on the 18 bp protospacer with one SNP, map-F2 (TATTTTTGAG**C**GCTTGTG) was added; Tube map-F3: sgRNA based on the 18 bp protospacer with one SNP, map-F3 (TATTTTTGAGTGCTTG**C**G) was added; Tube NC: only the 5′-^6^FAM-N12-3′-BHQ1 probe was added. **(B)** Fluorescence intensity corresponding to each tube in panel **(A)**. Paired two-tailed *t*-test, ^∗^*p* < 0.05, ^∗∗^*p* < 0.01, ^∗∗∗^*p* < 0.001. NS, Not significant. NC, Negative control.

Supplementary Figure 2 Feasibility assessment of the CRISPR-Cas12b-based *C. jejuni* detection system for onsite *C. jejuni* detection based on environmental and chicken samples collected from a retail market. **(A)** Fluorescence signals triggered by the 118 samples. Tube 1–118: crude DNA extracted from 118 samples was added; Tube 119: no DNA and probe were added; Tube 120: only the 5′-^6^FAM-N12-3′-BHQ1 probe was added; Tube 121: positive sample. **(B)** Comparisons between the cultivation-based and the CRISPR-Cas12b-based *C. jejuni* detection.

Supplementary Table 1 The *C. jejuni* genomes used for protospacer discovery.

Supplementary Table 2 The *C. coli* genomes used for protospacer discovery.
