# Supplementary figures and images for: Rapid and Accurate Campylobacter jejuni Detection With CRISPR-Cas12b Based on Newly Identified Campylobacter jejuni-Specific and -Conserved Genomic Signatures
Source: Front Microbiol. 2021 Apr 27;12:649010. doi: 10.3389/fmicb.2021.649010 (PMC8110837; doi:10.3389/fmicb.2021.649010)

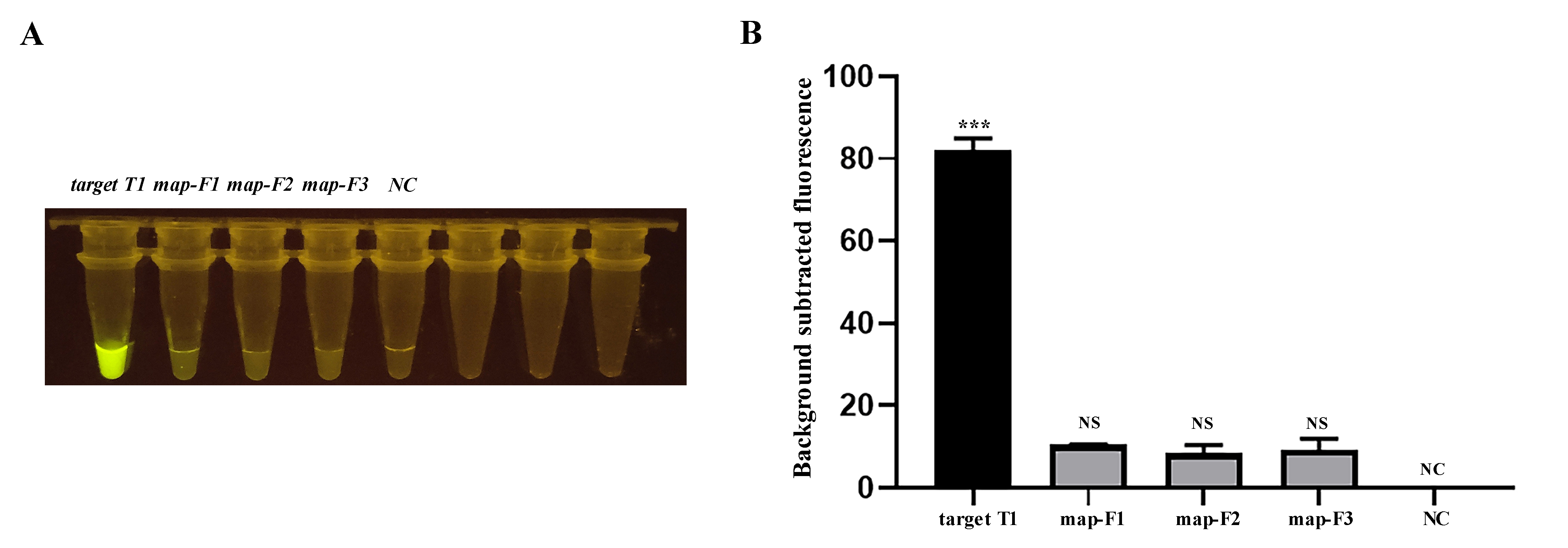

Supplement: Supplementary Figure 1 — Feasibility Assessment of the candidate protospacers identified from the mapA primer sequences. (A) Fluorescence signals in the CRISPR-Cas12b-based detection system when different candidate protospacers were added in the system. Tube target T1:sgRNA based on protospacer target T1 was added; Tube map-F1: sgRNA based on the 18 bp protospacer which was conversed in 889 genomes, map-F1 (TATTTTTGAGTGCTTGTG) was added; Tube map-F2: sgRNA based on the 18 bp protospacer with one SNP, map-F2 (TATTTTTGAGCGCTTGTG) was added; Tube map-F3: sgRNA based on the 18 bp protospacer with one SNP, map-F3 (TATTTTTGAGTGCTTGCG) was added; Tube NC: only the 5′-6FAM-N12-3′-BHQ1 probe was added. (B) Fluorescence intensity corresponding to each tube in panel (A). Paired two-tailed t-test, ∗p < 0.05, ∗∗p < 0.01, ∗∗∗p < 0.001. NS, Not significant. NC, Negative control. [file Image_1.JPEG]
